# Effects of Different Photoperiods during Incubation on Post-Hatch Broiler Performance and Stress Response

**DOI:** 10.3390/vetsci11090418

**Published:** 2024-09-09

**Authors:** Yasir Arslan Noor, Muhammad Usman, Usman Elahi, Shahid Mehmood, Muhammad Faisal Riaz, Ehsaan Ullah Khan, Kinza Saleem, Sohail Ahmad

**Affiliations:** 1 Department of Poultry Production, Faculty of Animal Production and Technology, University of Veterinary and Animal Sciences, Lahore 54000, Pakistan; 2 Faculty of Agriculture and Veterinary Sciences, Superior University, Lahore 55150, Pakistan; 3 Department of Poultry Science, Faculty of Veterinary and Animal Sciences, Muhammad Nawaz Shareef University of Agriculture, Multan 60000, Pakistan; 4 Department of Animal Nutrition, Faculty of Animal Production and Technology, University of Veterinary and Animal Sciences, Lahore 54000, Pakistan

**Keywords:** broiler, lighted incubation, photoperiod, subsequent performance

## Abstract

**Simple Summary:**

This research examined how light exposure during incubation affects broiler chickens after hatch. Incubating eggs were exposed to different light durations during incubation (from none to all the time). After hatching, chicks’ growth, stress levels, health, and immune response were checked. Results show that light during incubation did not affect most of these measures; however, chicks exposed to 20 h of light had heavier hearts. Overall, light during incubation does not seem to have a big impact on post-hatch performance.

**Abstract:**

This study evaluated the subsequent effect of photoperiods during incubation on post-hatch growth and stress response of commercial broiler chickens. A total of 875 Ross 308 broiler breeder (48 weeks of age) eggs were hatched using different durations (0, 4, 8, 12, 16, 20, and 24 h a day) of dichromatic light [green and red (495 to 750 nm); 2700 K; 250 lux; SUNJIE; China] throughout the whole period of incubation. A total of 50 0-day-old hatched straight run broiler chicks from each photoperiod during incubation were used to evaluate subsequent growth performance (feed intake, body weight, and feed conversion ratio); stress parameters (physical asymmetry, tonic immobility, and vocalization,); welfare traits (feather score and gait score); carcass traits (live weight, dressed weight, carcass yield, liver weight, gizzard weight, heart weight, abdominal fat weight, breast weight, and leg weight); and serum chemistry (globulin, total protein, cholesterol, glucose, and uric acid). There were no influences of photoperiod during incubation on post-hatch growth, stress parameters, welfare, and carcass traits. Heart yield was higher in birds incubated under 20 h light than in those from the 16 h light group. Incubation under different lighting durations also altered blood biochemical profile but did not influence serum globulin and cholesterol levels. It was concluded that under experimental conditions, incubation of broiler eggs under different lighting durations did not impact subsequent post-hatch performance (21–35 d).

## 1. Introduction

Chicken development and growth may be significantly improved if chickens are adequately incubated throughout embryogenesis, which can have a long-term impact on a bird’s health [1]. The incubation period has gained importance in recent years, as the incubation and brooding periods cover one-half of the total production cycle [2]. The rise of the commercial chicken industry would not have been possible without gradual upgrading and automation in incubator machines, which have helped to consistently generate a large number of day-old chicks with better hatching capacities than what is produced by the natural process [2]. Scientists are working on epigenetic adaptations for improvement of chicks’ capacity to hatch, develop, and respond to their new environment by tweaking the incubation circumstances [3], as these embryonic epigenetic alterations have helped develop temperature-sensitive birds [4]. Although advancements in developmental biology and embryology have played an important role in the improvement of the incubator machine, incubators still need to be improved for better hatchability and chick quality. Worldwide, research is being undertaken either by altering the incubator machine’s interior environment or by supplying embryos with varied stimuli during incubation [3].

When broiler chicks are incubating, it may be beneficial to provide light to help them adapt to their new environments [5]. On the second day of incubation, embryos respond to light [6]. According to research, lighting incubation chambers have also benefited avian wellbeing and provided overall economic advantages [7]. It was found that Light-Emitting Diode (LED) illumination during incubation reduces the stress on chicks and allows hatchlings to begin their lives with less dread [8]. As a result of hormonal changes [9] and an alignment of circadian cycles, stress levels decrease. Researchers experimented with various colors and wavelengths of light to find the right combination of time and intensity for stimulating embryos. Even so, several investigations yielded conflicting findings; many research studies employed a light intensity of 200–300 lux, which is thought to promote hatchability and reduce anxiety and susceptibility to stress [9,10]. Different light sources and light intensities during incubation have a stimulating effect in boosting hatchability and embryo development rate, reducing the growth stress of the embryo, and eventually improving adult bird performance [9,10,11]. It is realistic to provide illumination during incubation to enhance hatchery procedures and the wellbeing of the birds [9,11].

It used to be less common to employ light sources during the incubation phase. However, when new lighting technologies such as LED emerged, this practice became more common. Incubators may be lighted without altering their internal temperature by employing current LED lights. Such new lights are advantageous since they are less costly, long-lasting, and a better match for natural light than older models [12]. For the early stages of broiler chicks, monochromatic green light positively affects hatchability and development rate [13,14]. Utilization of monochromatic lights of blue-green and green-blue combinations revealed improvement in the growth performance of broiler chicks [15]. The use of red and blue monochromatic light during incubation can control the post-embryonic development of broiler chicks in different ways, with possible impacts for their growth and welfare during incubation [16]. Providing 16 h of green light gives better results in body weight, feed intake, and feed conversion ratio as compared to 8 h of dark environment during incubation [17]. Light has a good impact on early growth performance, as chicks incubated under 12 h of lighted incubation gained more body weight during the first 6 h after hatching as compared to complete darkness during incubation [18]. For the physiological growth, behavior, health, and welfare of the chicken, illumination is a significant external abiotic stimulus [1]. Monochromatic illumination is used to enhance the productivity traits and health of chickens. Monochromatic LED light is a possible method to increase chicken production [6].

The disease and death rates of chicks during the initial stages of life have become an issue, and strategy is required to improve the chicks’ environment (nutrition, brooding temperature, etc.). To minimize the number of diseased birds, one beneficial strategy is to improve the incubation methods to produce healthy birds that easily cope with farm environments [19]. Numerous studies reported the effects of monochromatic light durations during incubation on hatching traits and post-hatch growth of broiler chicks. However, the literature regarding the impact of dichromatic light durations during incubation on post-hatch performance, especially stress response and meat quality traits, is still silent and requires further investigations. In our previous study, green and red (dichromatic) light was provided to broiler breeder eggs during incubation, and positive effects were noted on hatching results and post-hatch performance [20]. Therefore, keeping in mind the impact of various regimes of light in terms of wavelength, intensity, and photoperiod, the current research study has been designed to investigate the influence of varying intervals of photo-stimulation (0, 4, 8, 12, 16, 20, and 24 h) during the incubation period on the hatching outcomes, post-hatch growth performance, carcass traits, and stress measures.

## 2. Materials and Methods

This experiment was a continuation of a previous study in the Department of Poultry Production, University of Veterinary and Animal Sciences, Ravi Campus, Pattoki, Pakistan. During the first phase, Ross 308 broiler breeder (48 weeks of age) eggs (n = 875) were exposed to different durations (0, 4, 8, 12, 16, 20, and 24 h a day) of dichromatic light [green and red (495 to 750 nm); 2700 K, 250 lux; SUNJIE, China] throughout the whole period of incubation, and the effect on hatching traits was evaluated. A total of 50 0-day-old hatched straight run broiler chicks from each photoperiod during incubation were used to evaluate subsequent growth performance, along with welfare aspects and blood chemistry up to 2 weeks post-hatch. Hatching eggs were incubated in an Italian-made incubator (Victoria Inc., Hong Kong, China, Quaglie I-36 and H-24) during the first 18 d; 37.5 °C temperature and 55% relative humidity were provided, with 8 turning times a day. During the last 3 d, 36.5 °C temperature and 65% relative humidity were provided.

This study evaluated the subsequent effect of a different duration of dichromatic light during incubation on post-hatch performance of broiler chicks in later stages (3rd, 4th, and 5th week) of life. In total, 350 straight run broiler chicks (Ross 308) were distributed across 7 treatment groups with 5 replicates of 10 birds each, according to a completely randomized design. The different durations of green and red light utilized during incubation were taken as treatments, which were 0, 4, 8, 12, 16, 20, and 24 h a day with intensity of 250 lux.

### 2.1. Bird’s Husbandry

Chicks were reared in an experimental poultry shed with measurements of 40 × 60 feet, providing a space of 0.06 m^2^ per bird. Birds were provided with rice husk as bedding material. The chicks were fed *ad libitum* corn soy-based diets according to the recommendation of the ROSS-308 manual (Ross Nutrition Specification) (Table 1), and water was provided via the nipple drinking system. Birds were vaccinated in accordance with the local area schedule (Table 2).

### 2.2. Parameters Evaluated

Data regarding growth performance were evaluated regarding feed intake, weight gain, and feed conversion ratio. 

### 2.3. Measures of Stress

Physical asymmetry: morphometric data of 5 birds per replicate were used to investigate physical asymmetry at the age of 35 d. A vernier caliper was used to measure the length and breadth of the metatarsals on both the left and right legs. Three qualities, metatarsal length (ml), metatarsal width (mw), and middle toe length (mtl), were used to compute the asymmetry of birds’ feet, which was then summed together and divided by the number of traits [21].

Composite asymmetry score = ({L-R (ml)} + {L-R (mtl)} + {L-R (mw)})/3
Middle toe length (mm) = mtl
Metatarsal length (mm) = ml
Metatarsal width (mm) = mw
No. of traits = 3

#### 2.3.1. Tonic Immobility

At the 5th week of age, tonic immobility was performed by removing all the birds (n = 10) out of a pen, transporting the birds to a specific area, and placing them in a container for 15 s. If a bird righted before 10 s, it was re-induced up to three times; if the bird could not be induced in three tries, it was scored as 0. Latency to right was recorded; if a bird failed to right within 120 s, it was given the maximum time score, and the test was terminated. This method was adapted from Archer and Mench [22].

#### 2.3.2. Vocalization

Chicks’ stress responses were assessed using an isolation test. All the birds (n = 10) were picked from a pen at 10 days of age, transferred to a remote site, and put in an uncovered plastic container for isolation testing. After that, these birds were placed in a 19 L bucket with no lid. During that time, a three-minute timer was set. The number of vocalizations made by the bird was counted. The bird was then transferred into the container specified for holding it. The ten birds were then returned to their enclosures after being tested, and ten birds from the next pen were removed and checked. Increased vocalizations were thought to indicate stress level of higher intensity [8].

### 2.4. Welfare Aspects

#### 2.4.1. Feather Score

There were 15 birds in each treatment at the ages of 15 and 35 days, and feather conditioning was graded on a point scale from 1 to 3. One signifies that the broiler chick is entirely feathered, two means that there are significant portions of the bird that are uncovered, and three means that there is no or very little feathering on the broiler chick [23].

#### 2.4.2. Gait Score

At the age of 35 days, the US gait score system was allocated to 105 birds (15/treatment) using the approach developed by Webster et al. [24]. Each bird’s gait was recorded and graded on a scale from 0 to 2, with 0 denoting perfect performance, 1 indicating evident symptoms, and 2 denoting more severe ones.

### 2.5. Carcass Characteristics

At the age of 35 d, 3 birds were picked at random from each replicate (15 birds from each treatment; total 105) and halal slaughtered (PS3733: 2016) to record pre-slaughter weight and carcass weight, as well as carcass, liver, gizzard, heart, abdominal fat, breast, and leg yield.

The organ yield percentage was computed using an eviscerated carcass with a neck but no skin or giblets.

Organ yield % = (Organ weight (g)) / (Live weight (g)) ×100

### 2.6. Serum Chemistry

Four birds per replicate were selected on day 35, and 3 mL of blood was collected to evaluate serum globulin, total protein (globulin and total protein were determined by using a total protein assay kit following the biuret method; Thermo Fisher Scientific, Shanghai, China), cholesterol (determined by cholesterol assay kit based on enzymatic colorimetric; Randox laboratories), glucose (determined by glucose assay kit based on glucose oxidase/peroxidase method; Sigma-Aldrich, Beijing, China), and uric acid (determined by uric acid assay kit based on Uricase method; Randox laboratories, Shanghai, China).

### 2.7. Statistical Analysis

Data regarding post-hatch performance were analyzed by using a one-way ANOVA technique [25]. GLM procedures were used in SAS software (version 9.1). Comparison of significant treatment means were computed through Tukey’s HSD test. The following mathematical model was applied:

Y_ij_ = µ + τ_i_ + ϵ_ij_

where

Y_ij_ = observation of dependent variable recorded on *i*th treatment group
µ = population mean
τ_i_ = effect of ith treatment group (i = 1, 2, 3, 4, 5, 6, 7)
ϵ_ij_ = residual effect of *j*th observation recorded on *i*th treatment group, NID~0, σ^2^

## 3. Results

**Figure 1:**
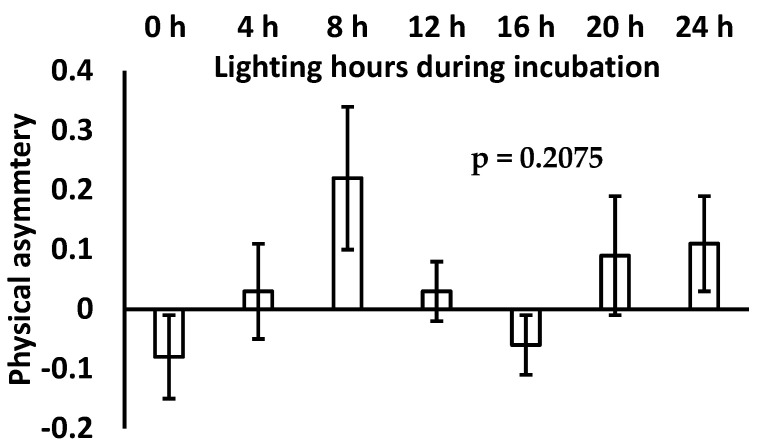
Physical asymmetry of commercial broiler among different treatment groups.

The influence of incubation lighting hours on physical asymmetry of broiler chicks is shown in Figure 1.

Incubation lighting hours showed no significant difference on physical asymmetry of broiler chicks; however, broiler chicks hatched under 8 h of incubation lighting showed higher numerical values for physical asymmetry. Nonetheless, broiler chicks hatched without and/or with 16 h incubation lighting showed numerically lower physical asymmetry values compared to all treatments. 

Broiler chicks hatched under different incubation lighting hours showed no significant difference on feed intake (Table 3). However, the 8 h incubation lighting group showed numerically higher values in the 3rd week. The 0 h group showed higher values in week 4, in week 5, and for total feed intake. Chicks hatched under various incubation lighting hours showed no significant difference in body weight (Table 3). However, the 24 h lighting group numerically increased body weight in week 3, the 4 h lighting group in week 4, and the 0 h group in week 5. Varied incubation lighting hours did not result in any significant difference in FCR of broiler chicks (Table 3). However, broiler chicks from 4 h, 16 h, and 20 h incubation lighting groups demonstrated numerically lower FCR for week 3 and lower cumulative FCR, while numerically lowered FCR was also observed in the 24 h group in week 3, in the 4 h and 20 h groups in week 4, and in the 0 h group in week 5. 

Stress parameters and welfare traits in broiler chicks were not affected by incubation lighting hours (Table 4). However, broiler chicks from the 4 h and 24 h incubation lighting group showed numerically shorter latency for tonic immobility. The 20 h group was the lowest for vocalization, the 16 h group was the lowest for feather score, and the 8 h and 24 h groups were lowest for gait score. 

Influence of incubation lighting hours on carcass characteristics of broiler chicks is shown in Table 5.

Chicks hatched with 20 h of light per day had a higher (*p* ≤ 0.05) heart yield than those hatched with 0, 4, and 16 h of light per day; however, the 16 h group exhibited a significantly (*p* ≤ 0.05) lower heart yield compared to other treatment groups. Nonetheless, no significant difference was observed in pre-slaughter weight, carcass weight, carcass yield, liver, gizzard, abdominal fat, breast, and leg yield; however, numerical differences were there. 

The impact of incubation lighting hours on serum chemistry of broiler chicks is shown in Table 6. Broiler chicks from the 16 h incubation lighting group exhibited a significantly higher (*p* ≤ 0.05) total protein level, the 20 h group increased (*p* ≤ 0.05) the glucose level, and the 12 h group increased (*p* ≤ 0.05) the uric acid level. However, total protein and glucose levels were reduced (*p* ≤ 0.05) in 4 h and 8 h incubation lighting groups; meanwhile, the 4 h lighting group reduced (*p* ≤ 0.05) the glucose level.

## 4. Discussion

The aim of the present study was to evaluate the effect of the photoperiod during incubation of broiler breeder eggs on subsequent (21–35 d) growth, welfare, stress response, and blood biochemistry. It was evident that light stimulation during embryogenesis may have influenced the development or hatching process but did not influence post-hatch performance of the birds. Only heart yield and serum chemistry parameters, e.g., total protein, glucose, and uric acid, exhibited differences among the treatments. 

Growth performance (feed intake, body weight, and feed conversion ratio) of broiler chickens was not influenced by photo-stimulation time (red and green light for 0, 4, 8, 12, 16, 20, and 24 h a day) during incubation, and this may be due to a complex environment after hatching that masks the effect of treatments. The response of the birds towards growth performance corresponds to the findings of Riaz et al. [26], who reported that broiler eggs exposed to white LED light during incubation for 12 h a day exhibited better feed conversion, whereas feed intake and weight gain did not differ among the birds provided with 24 h light and 24 h dark periods. Similarly, broiler eggs incubated under 12L:12D 24 h light (white LED) and 24 h dark revealed better feed conversion at 35 d post-hatch, whereas feed intake and weight gain were higher in the dark group [27]. Improved weight gain and feed conversion were also noted in Japanese quail that were provided with 24 h florescent light during incubation compared to a dark group [28]. However, another study reported that pre-hatch light (green and red) exposure to broiler eggs for 12 h a day did not influence post-hatch feed conversion [29]. 

This study suggested that welfare traits and stress response of broiler chickens were not influenced among the treatment groups (provision of red and green light for 0, 4, 8, 12, 16, 20, and 24 h a day). The most likely explanation for this response is that when broiler embryos were provided with lighted incubation, they responded to light during embryogenesis, and after hatching, birds were in a better position to respond to any photo stimuli that reduced their stress and improved their welfare traits. This corresponds to the findings of Archer and Mench [11], who suggested that provision of 12 h of light during incubation can reduce stress susceptibility in broiler chickens. Similar responses were reported by Yameen et al. [27]; feather score was similar in broiler chickens at 35 d in all the light regimens (12L:12D and 24L) during incubation and in the complete dark group.

In this study, carcass traits of broiler chickens did not differ among the treatment groups (red and green light for 0, 4, 8, 12, 16, 20, and 24 h a day). As the body weight of broiler chickens did not reveal differences at market age, carcass traits would therefore also be expected to be similar among the treatments. However, a contradictory study also reported higher breast and thigh yield in the 12 h lighted incubation group and higher abdominal fat in the 24 h lighted incubation group, whereas carcass yield was better in 12 h lighted incubation and complete dark groups [27]. In another study, a better carcass yield for broiler chickens was noted in 12L:12D and 24L groups compared to the complete dark group. Moreover, leg quarter yield was better in the 24L and complete dark group. However, breast and abdominal fat did not differ among 12L:12D, 24L, and complete dark groups [26]. 

It was surprising that blood biochemical profile differed among the treatment groups, where total protein, glucose, and uric acid were higher in the 16 h, 20 h, and 12 h light groups, respectively. However, in other studies, when Beijing You Chicken eggs were provided with different lighted regimes (24D, 8L:16D, 12L:12D, and 16L:8D) during incubation, it did not influence blood biochemical indexes (serum total protein, albumin, globulin, and urea nitrogen) [30].

## 5. Conclusions

It was concluded that under experimental conditions, lighted incubation with different durations for broiler eggs did not impact subsequent post-hatch performance (21–35 d). However, future studies may need to be conducted with a larger sample size. 

## Figures and Tables

**Table 1:** Nutrition specification for experimental birds.

		Starter	Grower	Finisher
Age Fed	Days	0–10	11–24	25–35
Energy per kg	Kcal	2975	3050	3100
Lysine	%	1.32	1.18	1.08
Methionine + cyst(e)ine	%	1.00	0.92	0.86
Methionine	%	0.55	0.51	0.48
Threonine	%	0.88	0.79	0.72
Valine	%	1.00	0.91	0.84
Isoleucine	%	0.88	0.80	0.75
Arginine	%	1.40	1.27	1.17
Tryptophan	%	0.21	0.19	0.17
Leucine	%	1.45	1.30	1.19
Crude protein	%	23.0	21.5	19.5
Total calcium	%	0.95	0.75	0.65
Available Phosphorus	%	0.5	0.42	0.36

**Table 2:** Vaccination schedule.

Sr. No.	Age	Vaccine	Company	Route of Administration
1.	1	ND + IB	HIPRA, Lahore, Pakistan	Oral
2.	6	ND Killed	HIPRA, Lahore, Pakistan	Injection
3.	10	IBD GM97	HIPRA, Lahore, Pakistan	Oral
4.	12	ND CLONE 79	HIPRA, Lahore, Pakistan	Oral
5.	21	ND CLONE	HIPRA, Lahore, Pakistan	Oral

**Table 3:** Growth performance of commercial broiler chicks among different treatment groups.

Treatment			Body Weight (g)		
		Week 3	Week 4	Week 5	
Lighting hours during incubation	0 h	985	1584.67	2211.33	
	4 h	1011.33	1670.00	2049.33	
	8 h	966.33	1596.00	2072.67	
	12 h	1011.00	1583.67	2111.67	
	16 h	1013.00	1634.67	2103.67	
	20 h	1014.33	1648.00	2108.00	
	24 h	1016.33	1549.33	1971.33	
SEM		6.71	14.77	23.44	
*p*-value		0.3370	0.3239	0.2086	
		Feed Intake (g)			Total Feed Intake (g)
Lighting hours during incubation	0 h	1153.33	2078.33	3210.33	6442.00
	4 h	1157.00	2063.00	3082.33	6302.33
	8 h	1170.00	2077.67	3141.00	6388.67
	12 h	1168.33	2066.33	3132.00	6366.67
	16 h	1158.33	2071.00	3086.33	6315.67
	20 h	1153.67	2027.67	3144.33	6325.67
	24 h	1158.67	2037.33	3000.00	6196.00
SEM		2.51	5.87	19.73	23.04
*p*-value		0.4565	0.0977	0.1144	0.1037
		FCR			Cumulative FCR
Lighting hours during incubation	0 h	1.17	1.31	1.45	1.31
	4 h	1.14	1.24	1.50	1.29
	8 h	1.21	1.30	1.52	1.34
	12 h	1.16	1.30	1.48	1.31
	16 h	1.14	1.27	1.47	1.29
	20 h	1.14	1.24	1.49	1.29
	24 h	1.14	1.32	1.52	1.33
SEM		0.01	0.01	0.01	0.01
*p*-value		0.2239	0.1688	0.4790	0.1711

**Table 4:** Stress parameters and welfare traits of commercial broiler chicks among different treatment groups.

Treatment		Tonic Immobility Latency to Right (s)	Isolation Test Vocalization (#/3min)	Feather Score	Gait Score
Lighting hours during incubation	0 h	138.33	53.33	1.67	1.67
	4 h	130.67	55.33	1.67	1.33
	8 h	151.00	54.67	1.67	1.00
	12 h	145.00	54.33	1.67	1.33
	16 h	152.00	54.33	1.33	1.67
	20 h	137.00	50.00	2.00	1.67
	24 h	130.00	62.33	1.67	1.00
SEM		4.39	1.28	0.16	0.15
*p*-value		0.7890	0.3114	0.9846	0.7874

**Table 5:** Carcass traits of commercial broilers among different treatment groups.

Traits	Lighting Hours during Incubation							SEM	*p*-Value
	0 h	4 h	8 h	12 h	16 h	20 h	24 h		
PSW, g	2171.4	2136.0	2171.3	2186.7	1948.0	2040.0	2034.0	28.27	0.175
CW, g	1468.4	1414.0	1481.3	1501.3	1324.0	1409.3	1375.3	21.52	0.329
CY %	67.6	66.2	68.1	68.7	68.0	69.1	67.6	0.33	0.443
LvY, %	1.96	2.01	1.86	1.95	2.17	2.13	2.04	0.06	0.866
GY, %	2.41	2.45	2.30	2.53	2.51	2.58	2.90	0.07	0.412
HY, %	0.38^c^	0.39^c^	0.45 ^bc^	0.41^c^	0.41^c^	0.52^a^	0.49 ^ab^	0.01	0.004
AFY, %	1.62	2.11	1.64	1.42	1.72	1.57	1.66	0.08	0.547
BrY, %	32.88	29.35	31.41	31.92	32.03	31.97	31.43	0.40	0.378
LgY, %	24.59	24.76	24.15	24.19	24.20	26.23	24.04	0.34	0.727

Superscripts on different means within rows differ significantly at *p* ≤ 0.05; PSW = pre-slaughter weight; CW = carcass weight; CY = carcass yield; LvY = liver yield; GY = gizzard yield; HY = heart yield; AFY = abdominal fat yield; BrY = breast yield; LgY = leg yield.

**Table 6:** Serum chemistry of commercial broiler chicks among different treatment groups.

Treatment		SG (g/dL)	TP(g/dL)	CHL (mg/dL)	GLU (mmol/L)	UA (mg/dL)
Lighting hours during incubation	0 h	1.48	3.01 ^ab^	120.65	15.81 ^ab^	2.91 ^b^
	4 h	1.19	2.50 ^b^	111.92	12.73 ^b^	2.25 ^c^
	8 h	1.67	2.52 ^b^	117.75	12.68 ^b^	2.78 ^b^
	12 h	1.79	3.17 ^ab^	128.93	13.54 ^b^	3.73 ^a^
	16 h	1.41	3.66 ^a^	129.70	15.24 ^ab^	2.81 ^b^
	20 h	1.78	2.93 ^b^	123.95	19.34 ^a^	2.97 ^b^
	24 h	1.65	3.04 ^ab^	121.85	15.86 ^ab^	2.98 ^b^
SEM		0.06	0.10	1.83	0.62	0.10
*p*-value		0.0744	0.0263	0.1422	0.0429	0.0010

Superscripts on different means within column differ significantly at *p* ≤ 0.05; SG = serum globulin; TP = total protein; CHL = cholesterol; GLU = glucose; UA = uric acid.

## Data Availability

Data is contained within the research article.

## References

[1] Archer G.S., Mench J.A. (2017). Exposing Avian Embryos to Light Affects Post-Hatch Anti-Predator Fear Responses. Appl. Anim. Behav. Sci..

[2] Hulet R.M. (2007). Symposium: Managing the Embryo for Performance Managing Incubation: Where Are We and Why?. Poult. Sci..

[3] Decuypere E., Bruggeman V. (2007). The Endocrine Interface of Environmental and Egg Factors Affecting Chick Quality. Poult. Sci..

[4] Yalçin S., Özkan S., Siegel P., Yenisey Ç., Akşit M. (2012). Manipulation of Incubation Temperatures to Increase Cold Resistance of Broilers:Influence on Embryo Development, Organ Weights, Hormones and Body Composition. J. Poult. Sci..

[5] Archer G.S., Shivaprasad H.L., Mench J.A. (2009). Effect of Providing Light during Incubation on the Health, Productivity, and Behavior of Broiler Chickens. Poult. Sci..

[6] Zhang L., Zhu X.D., Wang X.F., Li J.L., Gao F., Zhou G.H. (2016). Green Light-Emitting Diodes Light Stimuli during Incubation Enhances Posthatch Growth without Disrupting Normal Eye Development of Broiler Embryos and Hatchlings. Asian-Aust. J. Anim. Sci..

[7] Huth J.C., Archer G.S. (2015). Effects of LED Lighting during Incubation on Layer and Broiler Hatchability, Chick Quality, Stress Susceptibility and Post-Hatch Growth. Poult. Sci..

[8] Archer G.S., Mench J.A. (2014). The Effects of the Duration and Onset of Light Stimulation during Incubation on the Behavior, Plasma Melatonin Levels, and Productivity of Broiler Chickens. J. Anim. Sci..

[9] Özkan S., Yalçin S., Babacanoǧlu E., Kozanoǧlu H., Karadaş F., Uysal S. (2012). Photoperiodic Lighting (16 Hours of Light:8 Hours of Dark) Programs during Incubation: 1. Effects on Growth and Circadian Physiological Traits of Embryos and Early Stress Response of Broiler Chickens. Poult. Sci..

[10] Archer G.S. (2017). Exposing Broiler Eggs to Green, Red and White Light during Incubation. Animal.

[11] Archer G.S., Mench J.A. (2013). The Effects of Light Stimulation during Incubation on Indicators of Stress Susceptibility in Broilers. Poult. Sci..

[12] Benson E.R., Hougentogler D.P., McGurk J., Herrman E., Alphin R.L. (2013). Durability of Incandescent, Compact Fluorescent, and Light Emitting Diode Lamps in Poultry Conditions. Appl. Eng. Agric..

[13] Rozenboim I., Biran I., Chaiseha Y., Yahav S., Rosenstrauch A., Sklan D., Halevy O. (2004). The Effect of a Green and Blue Monochromatic Light Combination on Broiler Growth and Development. Poult. Sci..

[14] Ke Y.Y., Liu W.J., Wang Z.X., Chen Y.X. (2011). Effects of Monochromatic Light on Quality Properties and Antioxidation of Meat in Broilers. Poult. Sci..

[15] Cao J., Wang Z., Dong Y., Zhang Z., Li J., Li F., Chen Y. (2012). Effect of Combinations of Monochromatic Lights on Growth and Productive Performance of Broilers. Poult. Sci..

[16] Drozdová A., Kaňková Z., Bilčík B., Zeman M. (2021). Prenatal Effects of Red and Blue Light on Physiological and Behavioural Parameters of Broiler Chickens. Czech J. Anim. Sci..

[17] Güz B.C., Molenaar R., de Jong I.C., Kemp B., van Krimpen M., van den Brand H. (2021). Effects of Green Light Emitting Diode Light during Incubation and Dietary Organic Macro and Trace Minerals during Rearing on Tibia Characteristics of Broiler Chickens at Slaughter Age. Poult. Sci..

[18] Henry J. (2020). The Use of Led Light during Incubation on Hatching and Posthatch Performance for Distinct Chicken Lines.

[19] Ibrahim M.M.A., Nelson J.R., Archer G.S., Athrey G. (2021). Effects of Monochromatic Lighting During Incubation and Vaccination on the Splenic Transcriptome Profiles of Chicken. Front. Genet..

[20] Riaz M.F., Mahmud A., Hussain J., Saima (2024). Impact of dichromatic lighted incubation on hatching result and post-hatch performance of broiler chickens. Trop Anim. Health Prod..

[21] Archer G.S. (2016). Spectrum of White Light during Incubation: Warm vs Cool White LED Lighting. Int. J. Poult. Sci..

[22] Archer G.S., Mench J.A. (2014). Natural Incubation Patterns and the Effects of Exposing Eggs to Light at Various Times during Incubation on Post-Hatch Fear and Stress Responses in Broiler (Meat) Chickens. Appl. Anim. Behav. Sci..

[23] Ipek A., Sozcu A. (2016). The Effects of Eggshell Temperature Fluctuations during Incubation on Welfare Status and Gait Score of Broilers. Poult. Sci..

[24] Webster A.B., Fairchild B.D., Cummings T.S., Stayer P.A. (2008). Validation of a Three-Point Gait-Scoring System for Field Assessment of Walking Ability of Commercial Broilers. J. Appl. Poult. Res..

[25] Steel R.G., Torrie J.H., Dickey D.A. (1997). Dickey Principles and Procedures of Statistics: A Biometrical Approach|NHBS.

[26] Riaz M.F., Mahmud A., Hussain J., ur Rehman A., Usman M., Mehmood S., Ahmad S. (2021). Impact of Light Stimulation during Incubation on Hatching Traits and Post-Hatch Performance of Commercial Broilers. Trop Anim. Health Prod..

[27] Yameen R.M.K., Hussain J., Mahmud A. (2020). Saima Effects of Different Light Durations during Incubation on Hatching, Subsequent Growth, Welfare, and Meat Quality Traits among Three Broiler Strains. Trop Anim. Health Prod..

[28] Khalil H.A. (2009). Productive and Physiological Responses of Japanese Quail Embryos to Light Regime during Incubation Period. Slovak J. Anim. Sci..

[29] Riaz M.F., Bergman M., Schober J., Oluwagbenga E., Christensen K., Fraley G.S. (2024). Red-Green or Full-Spectrum White LEDs Have No Effects on Incubation or Post-Hatch Production Variables in Broilers. J. Appl. Poult. Res..

[30] Geng A.L., Zhang Y., Zhang J., Zeng L.C., Chang C., Wang H.H., Yan Z.X., Chu Q., Liu H.G. (2021). Effects of Light Regime on the Hatching Performance, Body Development and Serum Biochemical Indexes in Beijing You Chicken. Poult. Sci..

